# Clinical course of COVID-19 patients needing supplemental oxygen outside the intensive care unit

**DOI:** 10.1038/s41598-021-81444-9

**Published:** 2021-01-26

**Authors:** Ayham Daher, Paul Balfanz, Maria Aetou, Bojan Hartmann, Dirk Müller-Wieland, Tobias Müller, Nikolaus Marx, Michael Dreher, Christian G. Cornelissen

**Affiliations:** 1 Department of Pneumology and Internal Intensive Care Medicine, University Hospital RWTH Aachen, Pauwelsstrasse 30, 52074 Aachen, Germany; 2 Department of Cardiology, Angiology and Internal Intensive Care Medicine, University Hospital RWTH Aachen, Aachen, Germany

**Keywords:** Virology, Infectious diseases, Respiratory tract diseases

## Abstract

Patients suffering from CVOID-19 mostly experience a benign course of the disease. Approximately 14% of SARS-CoV2 infected patients are admitted to a hospital. Cohorts exhibiting severe lung failure in the form of acute respiratory distress syndrome (ARDS) have been well characterized. Patients without ARDS but in need of supplementary oxygen have received much less attention. This study describes the diagnosis, symptoms, treatment and outcomes of hospitalized patients with COVID-19 needing oxygen support during their stay on regular ward. All 133 patients admitted to the RWTH Aachen university hospital with the diagnosis of COVID-19 were included in an observational registry. Clinical data sets were extracted from the hospital information system. This analysis includes all 57 patients requiring supplemental oxygen not admitted to the ICU. 57 patients needing supplemental oxygen and being treated outside the ICU were analyzed. Patients exhibited the typical set of symptoms for COVID-19. Of note, hypoxic patients mostly did not suffer from clinically relevant dyspnea despite oxygen saturations below 92%. Patients had fever for 7 [2–11] days and needed supplemental oxygen for 8 [5–13] days resulting in an overall hospitalization time of 12 [7–20] days. In addition, patients had persisting systemic inflammation with CRP levels remaining elevated until discharge or death. This description of COVID-19 patients requiring oxygen therapy should be taken into account when planning treatment capacity. Patients on oxygen need long-term inpatient care.

## Introduction

Since December 2019, the novel coronavirus “severe acute respiratory syndrome coronavirus 2 (SARS-CoV-2)”, has been causing a rapidly spreading international outbreak^1^. Due to the different related clinical scenarios which vary from asymptomatic infection to multiorgan involvement and failure, to even death in worse cases^2–5^, this disease has been causing a substantial burden on healthcare systems worldwide at every level and especially on available resources. However, the epidemiological studies from different countries have shown that the majority of infected patients (> 80%) are asymptomatic or have mild symptoms, whereas about 14% of infected patients have a severe disease and need to be hospitalized^2–7^. Nevertheless, among hospitalized patients, the presence and severity of respiratory failure are usually the most important clues in making the decision about admitting to the intensive care unit (ICU) in order to provide ventilatory support (non-invasive or invasive ventilation) or to treat on the regular ward. The group of ICU admitted patients has been very well characterized, and therapeutic approaches regarding ventilatory support have been well established^8–11^. On the other hand, there is a group of patients which have hypoxemic respiratory failure, but still could be managed on regular ward with supplemental oxygen therapy. The characteristics of these patients have—to the best of our knowledge—not been well described so far.

We therefore describe the diagnosis, symptoms, treatment and outcomes of hospitalized patients with COVID-19 needing oxygen support during their stay on regular ward.

## Methods

The protocol for this study was approved by the ethics committee of the University Hospital Aachen, Germany (EK 080/20). All investigations were performed in accordance with the ethical standards laid down in the Declaration of Helsinki in its latest revision and all patients provided written informed consent; in case patients could not provide consent, written consultant advice and next of kin permission was obtained.

Previously we compared patients with and without ARDS regarding differences and outcome^7^. The current analysis primarily focusses on patients with hypoxemic respiratory failure (defined as peripheral oxygen saturation on pulse oximetry (SpO_2_) < 92% on ambient air), which were admitted to a regular ward. Demographic data, disease history, coexisting medical conditions, presence of chronic respiratory failure, smoking history, and medication history were recorded for all patients. Symptoms at admission and a detailed history of present symptoms were also documented. Patients were assessed for eligibility on the basis of a positive reverse-transcriptase-polymerase-chain-reaction (RT-PCR) assay for SARS-CoV-2 in a respiratory tract sample tested by the local diagnostic laboratory. Viral load was also determined using RT-PCR. The threshold value Ct represents the time point, at which the exponential phase of amplification begins, which therefore is inverse proportional to virus concentration in the material investigated and reflects the relative difference on a logarithmic scale. The threshold value of the sample gene < 20, was classified as high. Values > 30 were classified as low virus load, and values between ≥ 20 and < 30 as medium virus load.

Overweight was defined as BMI > 25 kg/m^2^ and obesity as BMI > 30 kg/m^2^. Diabetes or prediabetes was defined by clinical history, medication and HbA_1c_ values ≥ 6.5%, or ≥ 5.7 to < 6.5%, respectively.

Vital signs including SpO_2_ were measured at least two times per day and if clinically indicated and documented in the hospital electronic medical record system. The worst values in 24 h were depicted for analysis. Febrile days were defined as the time from fever onset until the last documented value above 38.5 ℃.

Generally, supplemental oxygen was given to target SpO_2_ values of > 94% and clinical relief in patients without risk of hypercapnia, and SpO_2_ values of 88–92% with clinical relief if there was a risk of hypercapnia. The flow rates of supplemental oxygen where documented two times per day and adjusted when needed. An oxygen saturation below 92% led to an increase by 1 L/min while a sO2 above 96% triggered a decrease in oxygen flow by 1 L/min. Compliance to this process was checked daily by the attending physicians and is part of the hospital’s quality management program.

Serum, plasma, and whole blood samples were obtained routinely at the time of admission in all patients as per standard of care. Further blood tests were analyzed regularly as indicated, therefore patient numbers vary between different time points in the figures. Radiological and further microbiological tests were performed based on clinical decision making.

Values are displayed as median with interquartile ranges or mean ± standard error of the mean.

## Results

57 consecutive patients being hospitalized outside the ICU on an isolation ward for SARS-CoV-2 pneumonia between February and April 2020 were included into the analysis. All of them needed supplemental oxygen. At the time of this analysis, 13 of the 57 patients were deceased (23%), while 42 (74%) had been discharged from the hospital and 2 (4%) were still hospitalized. 12 of the 13 non-survivors (92%) expressed their will for a limitation of therapy during hospitalization and therefore did not wish to be resuscitated, intubated or treated on the ICU.

### Patient characteristics

Baseline characteristics of all patients as well as the subgroups of non-survivors and survivors are summarized in Table 1. The median age (IQR) of the overall cohort was 72 (60–81) years, and 23% were women. Non-survivors were older compared to survivors (Table 1). All but one patient had comorbidities, which are, as well as concomitant medication, displayed in Table 2. Survivors and non-survivors exhibited a similar prevalence of arterial hypertension, pre-existing respiratory diseases or pre-existing heart diseases (Table 2).

**Table 1:** Baseline characteristics.

	Total (N = 57)	Non-Survivors (N = 13)	Survivors (N = 42)
**Characteristics**			
Age, years	72 [60–81]	81 [76–86]	65 [56–78]
Female sex	13 (23%)	2 (15%)	10 (24%)
**Initial symptoms**			
Fever	39 (68%)	9 (69%)	29 (69%)
Cough	34 (60%)	10 (77%)	23 (55%)
Dyspnea	25 (44%)	4 (31%)	21 (50%)
Fatigue	21 (37%)	3 (23%)	18 (43%)
Gastrointestinal Symptoms	17 (30%)	1 (8%)	16 (38%)
Diarrhea	13 (23%)	0 (0%)	13 (31%)
Emesis	3 (5%)	1 (8%)	2 (5%)
Nausea	9 (16%)	1 (8%)	8 (19%)
Tiredness	16 (28%)	1 (8%)	15 (36%)
Myalgia	12 (21%)	1 (8%)	11 (26%)
Loss of Taste	10 (18%)	1 (8%)	9 (21%)
Loss of Smell	9 (16%)	1 (8%)	8 (19%)
Headache	7 (12%)	0 (0%)	7 (17%)
Sore throat	4 (7%)	0 (0%)	4 (7%)
Angina pectoris	4 (7%)	0 (0%)	4 (10%)
Pharyngalgia	3 (5%)	1 (8%)	2 (5%)
Rhinorrhoea	2 (4%)	0 (0%)	2 (5%)
**Symptom onset to Hospitalization, days**	4 [0–7]	0 [0–2]	6 [1–7]
**Inpatient treatment**			
Patients with antibiotic therapy^1^	22 (39%)	9 (69%)	11 (26%)
Duration of antibiotic therapy, days	5 [4–6]	4 [4, 5]	5 [4–7]
COVID-19 specific treatment	–	–	–
**Periods, days**			
Fever days	7 [2–11]	8 [4–11]	8 [2–11]
Hospitalization	12 [7–20]	9 [6–15]	13 [8–20]
Oxygen supplementation	8 [5–13]	7 [4–10]	9 [5–13]
**Outcome**			
Survivor	42 (74%)	–	–
Non-Survivor	13 (23%)	–	–
Ongoing hospitalization	2 (4%)	–	–
**Discharge location**			
Home	39 (68%)	–	39 (93%)
Rehabilitation	0 (0%)	–	–
Hospice	0 (0%)	–	–
Nursing facility	3 (5%)	–	3 (7%)
Discharge with oxygen therapy	3 (5%)	–	3 (5%)

Data in N (%) or Median [IQR].

IQR, Interquartile range.

^a^Antibiotic classes most commonly used: aminopenicillines, cephalosporines.

**Table 2:** Comorbidities and premedication.

	Total (N = 57)	Non-Survivors (N = 13)	Survivors (N = 42)
**Comorbidities**			
Total	56 (98)	13 (100)	41 (98)
Arterial hypertension	33 (58)	10 (77)	21 (50)
Pre-existing heart diseases	22 (39)	5 (38)	15 (36)
Cardiovascular disease	15 (26)	4 (31)	11 (26)
Atrial fibrillation	11 (19)	4 (31)	6 (14)
Heart failure	12 (21)	2 (15)	8 (19)
Pre-existing respiratory disease	20 (35)	5 (38)	15 (36)
COPD	10 (18)	1 (8)	9 (21)
Obstructive sleep apnea syndrome	5 (9)	2 (15)	3 (7)
Bronchial asthma	5 (9)	2 (15)	3 (7)
Other pulmonary diseases	8 (14)	3 (23)	5 (12)
Smoking	19 (33)	2 (15)	17 (40)
Former smoking	8 (14)	2 (15)	6 (14)
Continued smoking	7 (12)	0 (0)	7 (17)
Overweight (BMI ≥ 25 kg/m^2^, < 30 kg/m^2^)	17 (30)	2 (15)	14 (33)
Obesity (BMI ≥ 30 kg/m^2^)	12 (21)	1 (8)	11 (26)
Diabetes mellitus	17 (30)	5 (38)	10 (24)
Prediabetes	10 (18)	3 (23)	7 (17)
Chronic kidney disease	10 (18)	3 (23)	6 (14)
Malignancy	10 (18)	3 (23)	6 (14)
Cerebrovascular disease	6 (11)	1 (8)	4 (10)
Chronic hepatitis	4 (7)	1 (8)	3 (7)
Peripheral arterial occlusive disease	3 (5)	1 (8)	2 (5)
Chronic liver failure	3 (5)	1 (8)	2 (5)
**Premedication**			
ACE-Inhibitors	20 (35)	6 (46)	14 (33)
Angiotensin-receptor blockers	14 (25)	2 (15)	11 (26)
Beta blocker	20 (35)	7 (54)	11 (26)
Calcium antagonists	15 (26)	2 (15)	12 (29)
Diuretics	29 (51)	10 (77)	17 (40)
Antidiabetics	13 (23)	4 (31)	8 (19)
Lipid-lowering agents	16 (28)	6 (46)	10 (24)
Antiplatelet therapy	19 (33)	5 (38)	14 (33)
Anticoagulants	11 (19)	4 (31)	6 (14)
Inhalation therapy	16 (28)	2 (15)	13 (31)
Inhaled Glucocorticoids	5 (9)	1 (8)	4 (10)
Systemic Glucocorticoids	5 (9)	1 (8)	4 (10)
Immunosuppressive therapy	4 (7)	0 (0)	3 (7)
NSAIDs	11 (19)	0 (0)	11 (26)
Antibiotics	11 (19)	5 (38)	5 (12)
Antiviral therapy	1 (2)	0 (0)	1 (2)

Data in N (%)

COPD, chronic obstructive pulmonary disease; BMI, body mass index; ACE, Angiotensin-converting enzyme; NSAIDs, nonsteroidal anti-inflammatory drugs.

### Clinical course and laboratory findings

**Figure 1:**
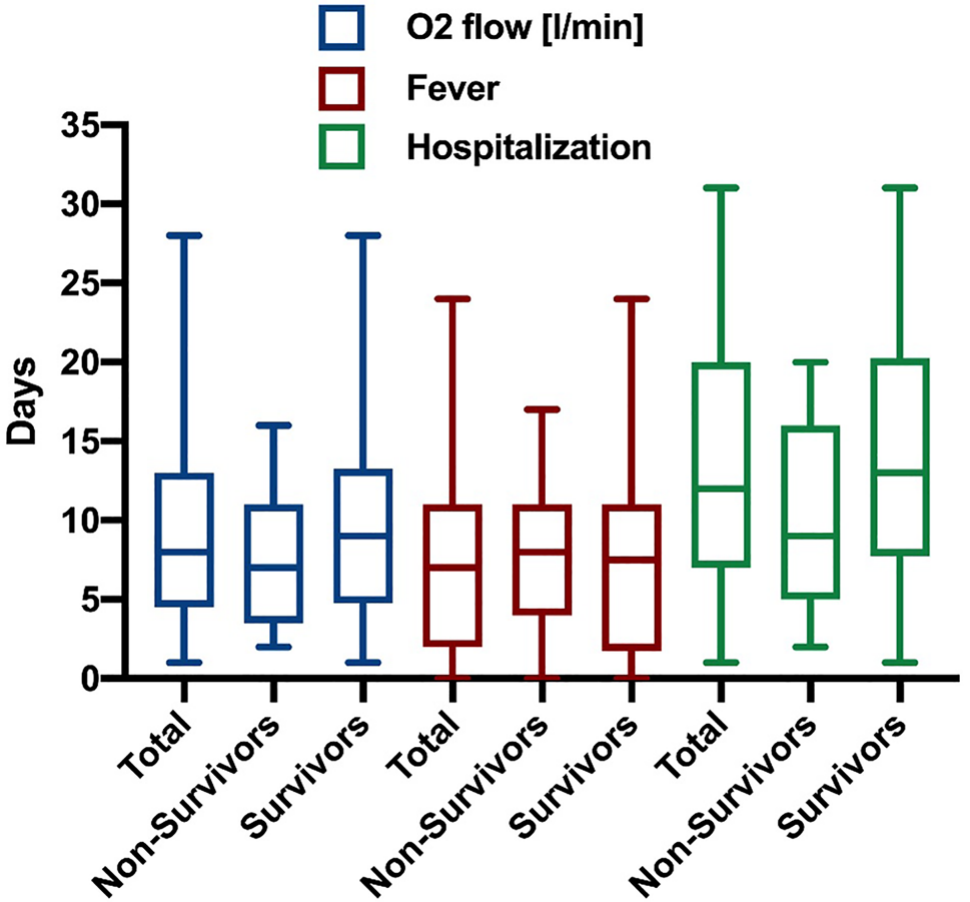
Clinical course of COVID-19 pneumonia. Box plots (Median with IQR and Min. to Max.) of oxygen supplementation (blue), fever (red) and hospitalization (green) each for total cohort, non-survivors and survivors.

**Figure 2:**
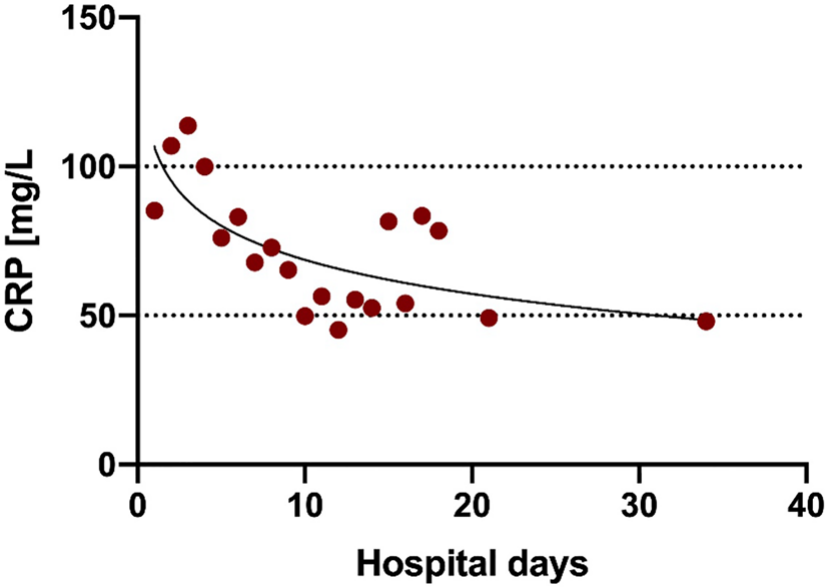
Example for the long-lasting elevation of inflammatory parameters in COVID-19 pneumonia. Mean of CRP (C reactive protein, red) in mg/L for complete cohort by hospital days.

47 (82%) patients reported fever as an initial symptom, whereas 24 (42%) patients had ongoing fever during hospitalization. Patients (survivors and non-survivors) had fever for 7 (2–11) days and needed supplemental oxygen for 8 (5–13) days resulting in an overall hospitalization time of 12 (7–20) days. Those being discharged had fever for 8 (2–11) days and needed supplemental oxygen for 9 (5–13) days resulting in an overall hospitalization of 13 (8–20) days (Fig. 1). In addition to the long period of hospitalization with fever and dependency on oxygen, inflammatory parameters were also elevated over a long time period (Fig. 2). In detail, 37 (65%) patients had CRP levels above 100 mg/L and 49 (86%) patients had CRP levels higher than 50 mg/L during hospitalization.

Vital signs as well as laboratory parameters, radiological and microbiological findings assessed at baseline are displayed in Tables 3 and 4. The median oxygen supply at admission was at 2 (0–4) L/min. 3 patients (5%) had already been on long-term oxygen therapy (LTOT). The median oxygen flow during the whole period of hospitalization was 2 (0.4–2.5) L/min. The majority of patients (77%) had bilateral infiltrates. Patients who died had higher values of inflammatory parameters compared to survivors (Table 3). In addition, D-dimers were elevated in all patients with higher values in those who died compared to survivors (Table 3).

**Table 3:** Vital and laboratory parameters.

	Reference values	Total (N = 57)	Non-Survivors (N = 13)	Survivors (N = 42)
**Vital parameters**				
Height, cm		172 [166–178]	173 [164–180]	173 [167–178]
Weight, kg		75 [66–80]	75 [66–80]	84 [72–91]
BMI, kg/m^2^		24.8 [23.5–27.2]	24.8 [23.5–27.2]	27.7 [24.2–30.3]
Respiratory rate, bpm		22 [18–26]	24 [17.5–28.3]	21 [18–25.8]
Oxygen saturation, %		94 [91–96]	94 [92–95]	95 [90.3–97]
Oxygen flow, l/min		2 [0–4]	3 [2–7]	2 [0–2]
Temperature, °C		37.9 [36.9–38.6]	37.8 [37–38.5]	37.9 [36.9–38.6]
sBP, mmHg		125 [101–140]	127 [110–140]	126 [103–140]
dBP, mmHg		71 [60–80]	67 [57–78]	74 [60–80]
Heart rate, bpm		88 [80–100]	88 [71–103]	90 [80–100]
**Laboratory tests at admission**				
Leukocytes, 1/nl	4.0–10.0	6.9 [5.1–9.5]	8.7 [5.9–9.3]	6.7 [4.5–9.4]
Hb, g/dl	m: 14.0–18.0	13.4 [10.3–14.5]	10.4 [10.1–13.5]	13.9 [12.1–14.6]
	w: 12.0–16.0			
Thrombocytes, 1/nl	150–400	180 [144–232]	148 [134–315]	187.5 [148.5–225.8]
Lymphocytes, %	22.0–53.0	10.4 [7–15.2]	7 [4.4–12.3]	11.6 [9.1–20.5]
INR		1.2 [1.1–1.3]	1.2 [1.1–1.4]	1.2 [1.1–1.3]
aPTT, sec	25.1 – 36.5	27.8 [26–30.7]	28.6 [26.1–30.9]	27.8 [26.2–30.4]
D-Dimer, ng/ml	< 500	855 [622–1086]	16,401 [11158–21644]	738.5 [595.8–881.3]
HbA1c, %	< 5.7	6.1 [5.5–6.7]	6.1 [5.8–6.8]	5.8 [5.4–6.6]
Sodium, mmol/l	136–145	139 [136–141.3]	141 [138–143]	139 [136–141]
Potassium, mmol/l	3.6–5.5	4.2 [3.8–4.6]	4.2 [3.6–4.7]	4.2 [3.8–4.6]
Albumin, g/dL	3.5–5.2	3.2 [2.7–3.8]	3.8 [3.6–3.9]	2.9 [2.7–3.8]
Total Bilirubin, mg/dL	< 1.2	0.6 [0.5–0.9]	0.9 [0.4–1.1]	0.6 [0.5–0.7]
AST, U/l	< 35	41.5 [30–55.3]	42 [27–72]	41 [33–55]
ALT, U/l	< 35	27 [20–37]	25.5 [20.8–29.5]	30 [20–41]
Gamma-GT, U/l	< 40	31 [23–68]	57 [32.5–110.8]	31 [23–65]
AP, U/l	35–105	65 [47–82.5]	98 [84–158.5]	61.5 [44.3–80.3]
LDH, U/l	m: 135–225	339 [277–442]	416 [332–452]	339.5 [273.3–436]
	w: 135–214			
CK, U/l	m: < 174	132 [82.8–285.5]	96 [62–400]	150.5 [98–285.5]
	w: < 140			
CK-MB-Activity, U/l	< 26 U/l	16 [11–18]	18 [14–18.5]	16 [12–18]
hsTroponin T, pg/ml	< 14.0	83.5 [27.3–131]	81 [68.5–93.5]	93 [28–179]
NTproBNP, pg/ml	< 220	726 [198.2–1393]	1730.5 [663.7–3028.3]	323.9 [71.5–1237]
Urea, mg/dl	16.6–48.5	40 [29.5–65.5]	48.5 [41–69.3]	38 [27–58]
Creatinine, mg/dl	0.5–1.2	1 [0.9–1.4]	1 [0.9–2]	1 [0.9–1.2]
CRP, mg/l	< 5	68.1 [29.9–119.8]	96.6 [31.3–135.4]	67 [28.2–109.9]
PCT, ng/ml	< 0.5	0.1 [0.1–0.2]	0.1 [0.1–0.3]	0.1 [0.1–0.2]
IL-6, pg/ml	< 7.0	64.2 [40.1–124.3]	697.8 [374.2–1021.4]	67.5 [42.1–123.6]
Ferritin, ng/ml	15.0—150.0	832.8 [455.6–1136.3]	221.7 [221.7–221.7]	1140.5 [1136.3–1144.8]

Data in Median [IQR]

IQR, interquartile range; BMI, body mass index; sBP, systolic blood pressure, dBP, diastolic blood pressure; Hb, hemoglobin; INR, international normalized ratio; aPTT, activated partial thromboplastin time; AST, aspartate aminotransferase; ALT, alanine aminotransferase; Gamma-GT, gamma-glutamyltranspeptidase; AP, alkaline phosphatase; LDH, lactate dehydrogenase; CK, creatine kinase; hs, high sensitive; NTproBNP, N-terminale pro brain natriuretic peptide; CRP, C-reactive protein; PCT, procalcitonin; IL-6, interleukin-6.

**Table 4:** Radiological and microbiological findings.

	Total (N = 57)	Non-Survivors (N = 13)	Survivors (N = 42)
**Chest radiography**			
No infiltrates	7/53 (13%)	3/13 (23%)	3/38 (8%)
Unilateral infiltrates	5/53 (9%)	2/13 (15%)	3/38 (8%)
Bilateral infiltrates	41/53 (77%)	8/13 (62%)	32/38 (84%)
**Viral load**			
High	11/52 (21%)	4/12 (33%)	7/38 (18%)
Medium	29/52 (56%)	7/12 (58%)	21/38 (55%)
Low	12/52 (23%)	1/12 (8%)	10/38 (26%)
Ct S-Gen	24,8 [2–29]	22,8 [2, 9–28]	24,1 [8–28]
**Viral detection**			
Respiratory detection	52/56 (93%)	12/13 (92%)	38/41 (93%)
positive out of hospital	7/57 (12%)	1/13 (8%)	6/42 (14%)
Extra-respiratory detection	11/34 (32%)	3/4 (75%)	8/28 (29%)
Serum	6/28 (21%)	2/4 (50%)	4/22 (18%)
Stool	5/15 (33%)	1/3 (33%)	4/12 (33%)
Urine	4/24 (17%)	2/4 (50%)	2/18 (11%)
**Bacterial detection**			
Blood culture	3/44 (7%)	1/12 (8%)	2/30 (7%)
Urine culture	16/38 (42%)	3/9 (33%)	11/27 (41%)

Data in N/total available N (%), or Median [IQR]

IQR, Interquartile range.

## Discussion

This study characterizes patients suffering from COVID-19 that require supplemental oxygen therapy but do not exhibit severe Acute Respiratory Distress Syndrome (ARDS) and can be treated outside the ICU. Several studies have focused on COVID-19 patients needing intensive care medicine^8–11^, but to our knowledge, patients requiring supplemental oxygen on a general ward have not been described in detail.

The patients included in this study presented with a typical set of symptoms^2^: fever, cough or fatigue were usually present. Of note, less than half (44%) of the patients exhibited dyspnea despite their hypoxemic respiratory failure, which might easily result in underestimation of the clinical severity of the disease. Peripheral oxygen saturation should thus be measured in all patients with COVID-19 at admission and routinely on regular basis during the hospital stay. All but one patient had at least one comorbidity; with hypertension, heart diseases and overweight being the most common ones.

Survivors and non-survivors in this study should be regarded as two different patient groups. While survivors were on average 16 years younger, non-survivors declined intensive care treatment. Respiratory failure led to death in the latter patients. Marked systemic inflammation reflected by IL-6 levels highlights the difference in disease severity between these groups. Also, an extra pulmonary manifestation of COVID-19 was detected in 75% of non-survivors, comparable to patients with mild ARDS^11^.

The single most outstanding finding of this study is the length of hospitalization and the need of supplemental oxygen: patients were treated for 12 days and needed oxygen therapy for 8 days on average. Hospital duration exceeded oxygen therapy whenever the overall patient status did not yet permit discharge or when home quarantine requirements could not be met. Importantly, comparing to patients being hospitalized because of severe influenza^12^, patients with COVID-19 seem to need a significantly longer hospital stay and are longer on oxygen therapy.

The severity and the prolonged course of COVID-19 in these patients might be caused by persisting systemic inflammation reflected in fever and elevated C-reactive protein (CRP) as well as interleukin-6 (IL-6). In fact, CRP levels remained elevated until discharge or death (ref. Figure 2).

In Conclusion patients with COVID-19 requiring oxygen therapy need long-term inpatient care with a median of 12 days in hospital including 8 days on supplemental oxygen, which should be taken into account when planning treatment capacity. This result could be partially explained by the prolonged inflammatory course of the disease.
